# Study of Predicting Longitudinal Cognitive Changes with “AN” Imaging Biomarkers in Subjective Cognitive Decline

**DOI:** 10.1002/alz70856_104224

**Published:** 2025-12-26

**Authors:** Shuhua Ren, Xiaoxie Mao, Qi Huang, Fang Xie, Yihui Guan

**Affiliations:** ^1^ Huashan Hospital, Shanghai, China; ^2^ School of Medicine, Xiamen University, Xiamen, Fujian, China; ^3^ Department of Nuclear Medicine & PET Center, Huashan Hospital, Fudan University, Shanghai, Shanghai, China; ^4^ Huashan Hospital, Fudan University, Shanghai, Shanghai, China; ^5^ Department of Nuclear Medicine and PET Center, Huashan Hospital, Fudan University, Shanghai, China

## Abstract

**Background:**

To explore whether baseline “AN” features in SCD, including brain Aβ deposition, hippocampal volume, and glucose metabolism in the hippocampus and parahippocampal gyrus, can predict longitudinal cognitive changes, thus identifying imaging biomarkers for SCD progression.

**Method:**

This study included 95 subjects with follow‐up (59 SCD patients and 36 normal controls). Baseline imaging included 18F‐Florbetapir PET/CT (brain Aβ), 18F‐FDG PET/CT (hippocampal and parahippocampal glucose metabolism), and T1‐weighted MRI (hippocampal volume). Cognitive function was assessed with neuropsychological tests at baseline and after two years. Cognitive change rates (Δchange = (follow‐up ‐ baseline) / baseline) were used as the primary outcome. A general linear model analyzed the relationship between baseline “AN” features and cognitive change rates, with Bonferroni correction for multiple comparisons.

**Result:**

Compared to the NC group, SCD subjects showed a trend toward accelerated decline in executive function (Shape Trail Test (STT)‐A score, β= ‐0.04, *p* = 0.083). Baseline whole‐brain Aβ deposition predicted longitudinal changes in the Boston Naming Test (BNT, β= ‐0.38, *p* = 0.00435) and STT‐A score (β= 0.25, *p* =  0.053). Baseline hippocampal volume predicted changes in Montreal Cognitive Assessment‐Basic (MoCA‐B, β= 0.43, *p* = 0.00449). Baseline FDG SUVr in the left parahippocampal gyrus predicted MoCA‐B (β= 0.36, *p* = 0.021) and STT‐B scores (β= ‐0.38, *p* =  0.0223). FDG SUVr in the right hippocampus predicted changes in BNT scores (β= 0.46, *p* =  0.00407).

**Conclusion:**

The 2‐year follow‐up indicated that SCD subjects experienced accelerated executive function decline. Baseline “A” and “N” imaging features predicted longitudinal cognitive changes in language, executive, and overall cognitive function in SCD. This study identifies baseline “AN” features as key imaging biomarkers for SCD progression.

